# Nebivolol combined with tetrahydrobiopterin affects diastolic function in spontaneously hypertensive rats via the nitric oxide/cyclic guanosine monophosphate signalling pathway

**DOI:** 10.1186/s40360-020-00460-z

**Published:** 2020-12-02

**Authors:** Xiaoli Guan, Xiaoying Guan, Changhong Lu, Bo Shang, Yuan Zhao, Ying Meng, Zhengyi Zhang

**Affiliations:** 1 General Medicine Department, Lanzhou University Second Hospital, Lanzhou, Gansu China; 2 Pathology Department, Lanzhou University Second Hospital, Lanzhou, Gansu China

**Keywords:** Tetrahydrobiopterin, Nebivolol,Hypertension, Rat, Diastolic function, Nitric oxide/cyclic guanosine monophosphate pathway

## Abstract

**Background:**

Hypertension is the the primary cause of diastolic heart failure. Oxidative stress plays an important role in cardiac diastolic dysfunction caused by hypertension. The occurrence of oxidative stress is related to the level of nitric oxide (NO) in the body. Tetrahydrobiopterin (BH4) is an essential cofactor for NO synthesis. Nebivolol can reduce myocardial oxidative stress and increase NO activity. Therefore, we investigated the effects of monotherapy or combination therapy of different doses of BH4 and nebivolol on cardiac diastolic function in spontaneously hypertensive rats, and preliminarily expounded the related mechanisms.

**Methods:**

Left ventricular function was evaluated by non-invasive echocardiographic assessment and invasive right carotid artery catheterization methods. ELISA was used to measure myocardial 3-nitrotyrosine content, NO production, and cyclic guanosine monophosphate (cGMP) concentration in the myocardium; quantitative real-time PCR (qRT-PCR) was used to determine endothelial nitric oxide synthase (eNOS), phospholamban and sarcoplasmic reticulum Ca^2+^-ATPase 2a (SERCA2a) mRNA expression levels; Western blot was used to detect the protein expression levels of eNOS and eNOS dimers in myocardial tissue, and immunohistochemical detection of cGMP expression in the myocardium was performed.

**Results:**

Studies have shown that compared with those in the control group, NO generation and the expression level of myocardial eNOS mRNA, eNOS expression of dimers, phospholamban, SERCA2a and cGMP increased significantly after the combined intervention of BH4 and nebivolol, while the expression of 3-nitrotyrosine was significantly decreased.

**Conclusions:**

The combined treatment group had a synergistic effect on reducing myocardial oxidative stress, increasing eNOS content, and increasing NO production, and had a more obvious protective effect on diastolic dysfunction through the nitric oxide/cyclic guanosine monophosphate (NO/cGMP) pathway.

**Supplementary Information:**

The online version contains supplementary material available at 10.1186/s40360-020-00460-z.

## Background

Hypertension is the most common chronic non-communicable disease and the most important risk factor for cardiovascular disease. It is known as the silent killer [1]. Hypertensive patients have a high incidence of heart damage, and its early manifestation is diastolic heart dysfunction, which has increasingly attracted the attention of scholars and experts. Diastolic dysfunction is an early manifestation of diastolic heart failure [2].

Diastolic dysfunction is an evolving clinical syndrome, and the contradiction between the increasing incidence and lack of effective clinical diagnosis and treatment options needs to be resolved urgently [3]. It is very urgent for medical researchers to conduct in-depth research on the prevention and treatment of diastolic dysfunction caused by hypertension. It is particularly important to improve the prognosis or prevent further deterioration of these patient groups.

Studies have shown that changes in cardiac diastolic function are related to sarcoplasmic reticulum Ca2+ and phospholamban [4]. The decrease in left ventricular relaxation is mainly related to the decreased function or decreased activity of SERCA2a, while SERCA2a activity and function are regulated by phosphorylation and dephosphorylation of PLN. Increasing evidence shows that oxidative stress plays an important role in the occurrence and development of hypertension, and is also an important factor leading to endothelial injury and dysfunction of target organs of hypertension. The occurrence of oxidative stress in hypertension is well-known to be related to NO [5]. NO has a protective effect on the cardiovascular system, which is necessary for the maintenance of diastolic function, and the synthesis of NO requires NOS, especially the enzymatic action of normal eNOS. By activating the soluble guanylate cyclase-cyclic guanosine phosphate pathway, NO can induce vasodilation and simultaneously has anti-inflammatory and anti-apoptotic effects and prevents myocardial function and structure disorders.

BH4 is a necessary cofactor for nitric oxide synthase to synthesize NO. When BH4 is lacking, it can accelerate eNOS decoupling, generate ROS instead of NO, and put the myocardium in an oxidative stress state. Long-term continuous oxidative stress can lead to impaired diastolic function [6]. At present, preliminary studies on the use of BH4 alone for cardiac diastolic function have shown that it can protect endothelial function and improve cardiac diastolic function [7, 8]. These studies are promising and controversial and have not been applied clinically for cardiovascular disease. Nebivolol is a third-generation β-adrenergic receptor (β-AR) blocker. Compared with other β-AR blockers, it has no intrinsic sympathomimetic activity [9]. Nebivolol has a unique pharmacological effect on the myocardium against oxidation and reduces oxidative stress, blocking β1-AR and β3-AR.

Therefore, according to the current antihypertensive treatment of a combination of drugs or a compound monotherapy treatment plan having an increased effect and reduced side effects, our study combined nebivolol with BH4. The purpose of this study was to investigate the effects of nebivolol and BH4 on myocardial oxidative stress and NO/cGMP pathway signalling molecule expression levels, thereby confirming whether it has a protective effect in diastolic dysfunction in spontaneously hypertensive rats (SHRs), determining whether it has a synergistic effect, and preliminarily clarifying its related mechanism.

## Methods

### Animals

Sixty 12-week-old male SHRs and 10 normal blood pressure Wistar-Kyoto (WKY) rats of the same age, weighing 230-260 g, were purchased from Beijing Vital River Laboratory Animal Technology Co., Ltd. (Beijing, China). The rats were fed at Lanzhou University Second Hospital Experimental Animal Center (SPF level), and were kept in polypropylene cages according to groups, with 4 to 6 rats in one cage. The feeding environment was 22 ± 1 °C and 55 ± 5% humidity, free access to standard feed and water, 12 h light/dark cycle. After the study, rats were sacrificed under anesthesia with 1% pentobarbital sodium (40 mg/kg) injection. The experiment was approved by the Medical Ethics Committee of Lanzhou University Second Hospital (Approval number: D2018–32). All animal experiments were carried out in accordance with Guidelines for Ethical Review of Laboratory Animal Welfare of China. Tetrahydrobiopterin (mass fraction was over 99%) was purchased from Guangzhou Kuwei Biological Technology Co., Ltd. (Guangzhou, China). Nebivolol was purchased from STADA Arzneimittel AG (Bad Vilbel,Germany).

Experimental grouping: A: WKY rats normal blood pressure control group (*n* = 6); furthermore, a total of 60 12-week-old male SHRs (animal models with increased blood pressure) were randomly (randomized block design) divided into groups (*n* = 6 in each group): B: SHRs baseline group, C: SHRs placebo control group (0.25 mL physiological saline with a 1 mL syringe), D: high-dose BH4 treatment group (administered with a 1 mL syringe, concentration was 70 mg/mL, dose was 25 mg/kg/d), abbreviated as H-BH4, E: low-dose BH4 treatment group (administered with a 1 mL syringe, concentration was 70 mg/mL and the dose was 12.5 mg/kg/d), abbreviated as L-BH4, F: high-dose nebivolol treatment group (administered with a 1 mL syringe, concentration: 5 mg/mL, dose: 5 mg /kg/d), abbreviated as H-N, G: low-dose nebivolol treatment group (administered with a 1 mL syringe, concentration: 5 mg/mL, dose: 2.5 mg/kg/d)), abbreviated as L-N, and H: combined drug treatment group, consisting of four groups: high-dose nebivolol (5 mg/kg/d) + high-dose BH4 treatment group (25 mg/kg/d), abbreviated as H-N & H-BH4; high-dose nebivolol (5 mg/kg/d) + low-dose BH4 treatment group (12.5 mg/kg/d), abbreviated as H-N & L-BH4; low-dose nebivolol (2.5 mg/kg/d) + high-dose BH4 treatment group (25 mg/kg/d), abbreviated as L-N & H-BH4; low-dose nebivolol (2.5 mg/kg/d) + low-dose BH4 treatment group (12.5 mg/kg/d), abbreviated as L-N & L-BH4.

### Non-invasive evaluation of cardiac function

The SHRs and WKY rats in each group were measured at baseline, and a non-invasive assessment of cardiac function was performed with cardiac ultrasound 1 week after treatment, with intraperitoneal injection of 2% pentobarbital sodium 0.3 mL/100 g prior to the measurement. Cardiac colour ultrasound (Vivid E9, Probe i13L Intraoperative Linear Probe, GE, USA) was used to measure cardiac function, and the probe frequency was 10–13 MHZ. M-mode echocardiography on the long axis of the left margin of the sternum and the horizontal axis of the papillary muscle were used to determine left ventricular posterior wall thickness, left ventricular end systolic dimensions and left ventricular end diastolic dimensions. The mean value of three consecutive cardiac cycles was taken as the measured value. Left ventricular ejection fraction and left ventricular fractional shortening were measured simultaneously.

Conventional pulse Doppler apical four-chamber view was used to measure early diastolic transmitral velocity (E) and lately diastolic transmitral velocity (A). Pulse Doppler was used to measure the velocity of the mitral annulus during early diastole (E’) and the velocity of the mitral annulus during late diastole (A’) through the apical four-chamber view of the mitral valve septum. Colour Doppler ultrasound was performed by the Colour Doppler Center at the Cardiology Department of Lanzhou University Second Hospital. The results were determined by blinded method.

### Invasive cardiac function test

After the SHRs and WKY rats baseline group completed the invasive cardiac function test, nine experimental groups (placebo control, high-dose and low-dose BH4 treatment group, high-dose and low-dose nebivolol treatment group, and four combination drug treatment groups) were subjected to the invasive cardiac function test 1 week after treatment. After anaesthesia, tracheostomy was performed, and animal ventilator assisted ventilation was performed (dhx-150, Chengdu Instrument Factory, respiratory frequency 60–80 times/min, tidal volume 2 mL/100 g, inhalation ratio 1:1). After respiration was stable, the animals were intubated through the carotid artery into the left ventricle and connected to the multilead physiological recorder (Millar Instruments, SPR-869, TX, USA) to determine the key indicators of left ventricular diastolic function: the maximum pressure rising rate and the maximum pressure decrease rate (± dp/dt max), the left ventricular systolic pressure, left ventricular end diastolic pressure and time constant of isovolumic LV relaxation.

### Determination of the concentration of different types of biopterin by high performance liquid chromatography

High performance liquid chromatography is currently one of the most widely-accepted methods for determining BH4 and BH2 levels [10]. The specific conditions and operating procedures are carried out according to the previously described [11]. Under optimized experimental conditions, BH2 and BH4 standard working solutions were obtained, and the levels of the target components, namely BH4 and BH2, were determined according to the chromatographic conditions explored in the preliminary experiment. Each sample was injected three times and the mean value was determined to generate a standard curve. Then the samples were detected by high performance liquid chromatography under a fluorescence detector (excitation wavelength 350 nm, emission wavelength 450 nm). The levels of BH4 and BH2 in the samples were calculated by the standard curves of BH4 and BH2 in a linear range.

### mRNA levels of eNOS, PLN and SERCA2 were detected by qRT-PCR

Total RNA was subsequently extracted with TRIzol agent (Invitrogen, Carlsbad, CA, USA) as per the manufacturer’s instructions. Experiments were independently repeated three times. A DNA synthesis kit (Takara, Kyoto, Japan) was used to synthesize cDNA from total RNA according to the manufacturer’s instructions. To detect the mRNA level of eNOS, PLN and SERCA2, quantitative real-time assays were performed on a LightCycler 480 system (Roche, Germany) with TB Green qPCR Master Mix (Takara, Kyoto, Japan). Primer sequences are shown in Table 1. The PCR procedures were as follows: Pre denaturation at 95 °C for 30 s, and 40 cycles of 95 °C for 5 s and 60 °C for 20 s. Melting curve analysis was performed at 95 °C 0 s, 65 °C15 s and 95 °C 0 s. Relative mRNA expression was calculated using the 2-ΔΔCt method with the ACTB gene as an internal control [12].

**Table 1:** Primer sequences of each target gene

Primer	Sequences
eNOS-F	GTGCTGGCATACAGAACCCA
eNOS-R	CCTGCCTTGAGTTGGCTCAT
SERCA2a-F	GTTCTGCTGCACAGTAGGGAT
SERCA2a-R	AGGCCAGCAGAAACTTGAGTAA
Phospholamban -F	TGGTGAATGGTCTGCGGAAT
Phospholamban -R	CAGGCGCTTTTCACCTTCCT
ACTB-F	AGTACAACCTTCTTGCAGCTCCTC
ACTB-R	TGCCGGAGCCGTTGTCG

### 3-NT and cGMP were determined by ELISA

After cutting the frozen myocardial specimens, 50 mg samples were weighed. The samples were stored at 2–8 °C after melting. A certain amount of PBS (pH 7.4) was added and the samples were fully homogenized by a homogenizer. Centrifugation was performed at 2000–3000 g/min for 20 min. Standards were diluted and added according to the kit instructions, and three replicates for each sample were set up. The samples were incubated at 37 °C for 30 min. In addition to the blank control wells, 50 μL of the enzyme-labelled antibody reagent was added to each well of the experimental samples, and the plate was then incubated and washed. The colour reaction was then developed and terminated. Finally, the absorbance value (OD value) of each well was measured with a microplate reader (BioTek, Winooski, VT, USA) at a wavelength of 450 nm, and the linear regression equation was listed according to the concentration and OD value of the standard substance. The OD value was substituted into the equation and then multiplied by the dilution factor to calculate the actual concentration of the sample.

### eNOS expression was detected by western blot

Fifty milligram rat myocardial tissue was weighed, placed into liquid nitrogen for repeated freezing-thawing, and then ground quickly by hand. Then, 200–300 μL protein lysate was added to lyse on ice. Immunoblot analyses of protein extracts were performed as described [13]. The membranes were blocked with 5% BSA in TBST and incubated with primary antibodies against eNOS (1:1000 dilution, Abcam, Cambridge, MA, USA) and GAPDH (1:1000 dilution, Abcam, Cambridge, MA, USA). Blots were then incubated for 1 h at 37 °C with goat anti-mouse or anti-rabbit secondary antibody (1:10000 dilution, Proteintech, Wuhan, China), and intensities were measured using enhanced chemiluminescence (Thermo Scientific, Waltham, MA, USA).

### Detection of eNOS dimers

The cells were lysed with cold cell lysis buffer (Sigma-Aldrich, St. Louis, MO, USA) mixed with a protease inhibitor (Roche, Germany). The lysates were then centrifuged at 12000 r/min at 4 °C for 5 min.The samples were prepared with Laemmli sample buffer (Roche, Germany). Then, eNOS dimers were detected by SDS-PAGE, and the lysed protein was separated under reducing conditions in an 8% Tris-glycine gel (Invitrogen, Carlsbad, CA, USA). All gels and buffers were pre-balanced before 4 °C electrophoresis and kept below 15 °C during electrophoresis. Incubation with a rabbit polyclonal antibody against eNOS (1:1000 dilution, Abcam, Cambridge, MA, USA) was performed as described in the previous section.

### eNOS activity detection

The principle of NOS activity detection is mainly based on NOS catalysing the biochemical conversion of L-arginine to L- citrulline to generate NO. Nitrogen on the L-arginine guanidyl group is oxidized to form NO while generating the corresponding stoichiometric L- citrulline [14]. The standard method for measuring NOS activity in natural or purified enzyme preparations is to determine the conversion of arginine to citrulline. Because the sensitivity of the radioactive substrate (3H arginine or 14C arginine) is at the pmol level, NOS activity can be calculated by quantitatively determining the amount of radioactive material in the eluate [14]. The specific experimental procedure was performed according to the eNOS activity detection kit manufactured by Cayman Chemical Company (Michigan, USA), and the measurement was conducted at the Nuclear Medicine Center of Lanzhou University Second Hospital and the Institute of Radiochemistry and Nuclear Environment, College of Nuclear Science and Technology of Lanzhou University.

### Immunohistochemical detection of cGMP in myocardial tissue

The embedded and fixed tissues were cut into 4-5 μm slices. Immunohistochemical detection was performed as described previously [15]. The slices were incubated with a primary antibody against cGMP (rabbit anti-rat, 1:300, CST, Beverly, MA, USA) at 4 °C overnight, After washing with PBS, an the slices were incubated with secondary goat anti-rabbit (HRP) IgG antibody (1:2000; Zhongshan Jinqiao, Beijing, China). Finally, the images were obtained by microscopy (Olympus, Tokyo, Japan).

### Statistical analysis

GraphPad Prism 7.0 software (GraphPad Software, Inc., La Jolla, CA, USA) was used to perform all the statistical analyses. The differences between two groups were evaluated by Student’s t-test, while comparisons of three or more groups were conducted by one-way analysis of variance (ANOVA) or two-factor analysis of variance followed by post hoc test. All values are the mean ± standard deviation of at least three independent experiments. *P* < 0.05 or *P* < 0.01 were considered to indicate a statistically significant difference.

## Results

### Relative diastolic dysfunction in SHRs

Compared with male WKY rats of the same age (Blood pressure: 121.16 ± 6.70, Heart rate: 370.66 ± 20.04), SHRs had significantly higher systolic blood pressure (207.66 ± 14.12) and heart rate (409.66 ± 47.19, *P* < 0.05, Table 2). The non-invasive diastolic function index E/A ratio was significantly lower (*p* = 0.014), the E/E’ ratio was significantly higher (*P* < 0.05, Table 2), and the left ventricular posterior wall thickness at end-diastole was thicker (*p* = 0.012). The time constant of isovolumic LV relaxation (tau), an indicator of invasive diastolic function, was significantly prolonged (*p* < 0.01). Compared with WKY rats, baseline SHRs at 12 weeks had higher left ventricular end-diastolic pressure (LVEDP) (*p* < 0.01).

**Table 2:** Comparison of basic indicators of WKY and SHR groups

Indexs	WKY(*n* = 6)	SHR(*n* = 6)	P
Body weight, g	251.06±12.70	246.78±5.32	0.78
Noninvasive measure			
Systolic blood pressure, mm Hg	121.10±6.56	207.66±14.12	0.001
Heart rate, bpm	370.66±20.04	409.66±47.19	0.030
Diastolic measures			
Peak early diastolic LV filling velocity	708.2±6.52	702±11.39	0.318
Peak E and A velocity ratio	2.03±0.12	1.55±0.08	0.014
E/E’	20.25±0.62	22.67±0.92	0.001
E’/ A’	1.58±0.23	0.86±0.02	<0.001
Systolic measures			
Fraction shortening, %	40.65±0.24	41.65±0.93	NS
Ejection fraction, %	74.63±0.38	75.86±1.03	NS
LV dimensions			
LV posterior wall thickness, mm	1.26±0.08	1.37±0.02	0.014
LV end-diastolic dimension, mm	6.03±0.03	6.04±0.05	NS
Invasive measure			
Heart rate	342.67±47.11	382.56± 46.05	0.025
Systolic function			
Max LV systolic pressure	101.55 ±4.64	146.60±11.29	0.001
dP/dtmax, mm Hg/s	6344.01±1744.84	7975.70±1434.87	0.09
Diastolic function			
LV end-diastolic pressure	11.92± 5.94	51.64±14.96	0.001
dP/dtmin,mm Hg/s	-4258.06±375.55	-6299.40±1192.38	0.001
dP/dtmin/LVSP	-49.17±6.60	-38.19±5.62	0.004
Time constant of isovolumic LV relaxation	18.51±3.35	25.9±3.38	0.001

Notes: Values are mean ± SD. NS means the difference was not statistically significant. A: Peak late diastolic LV filling velocity; E: Peak early diastolic LV filling velocity; E’and A’: Maximal velocity of mitral annulus during early and late diastole, LV: Left ventricle; LVSP: LV systolic pressure; SHRs: Spontaneously hypertensive rats, WKY: Wistar-Kyoto rats

### BH4 and nebivolol can synergistically improve diastolic dysfunction in SHR*s*

**Fig. 1:**
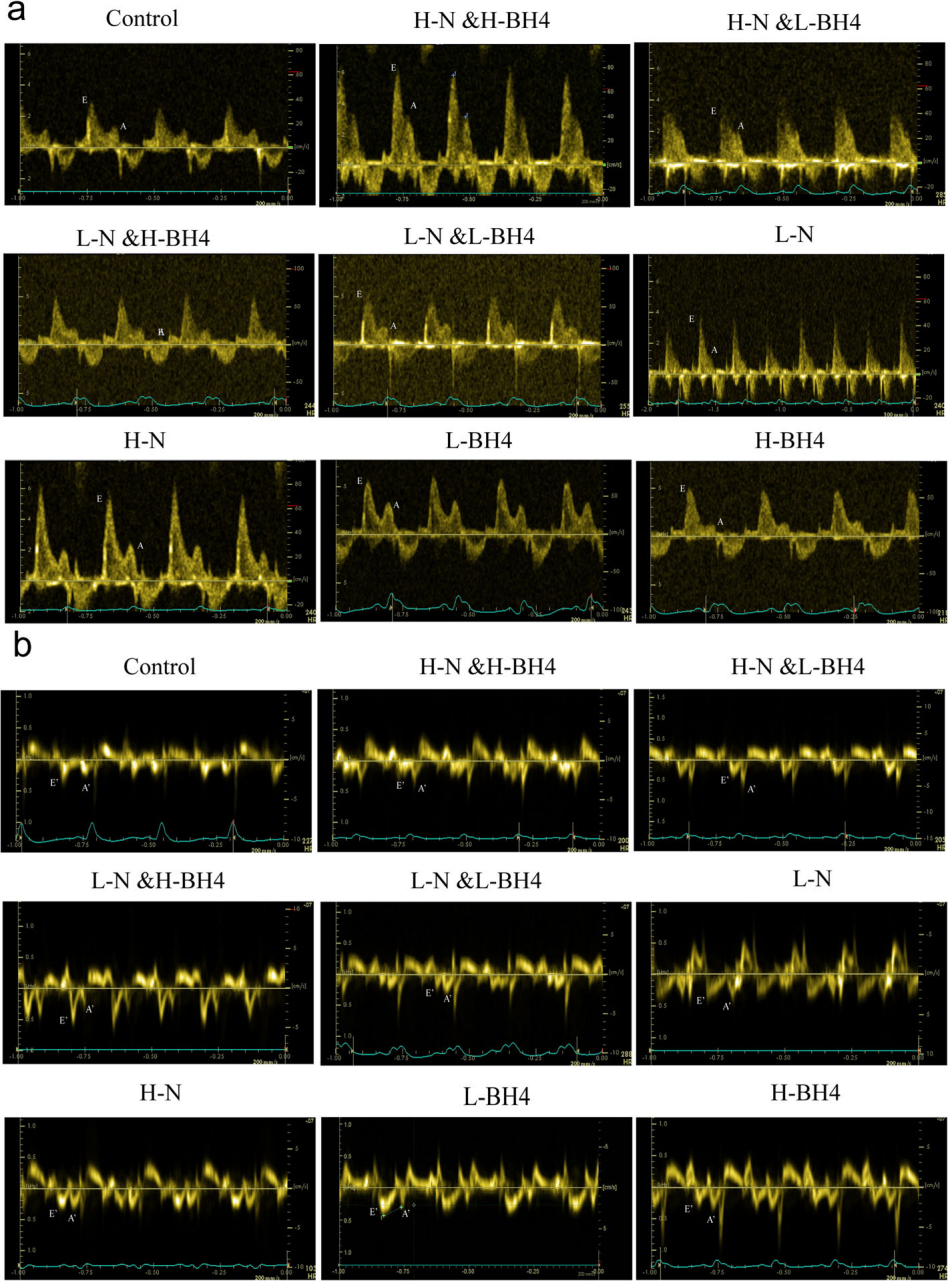
Comparison of cardiac function by colour ultrasound. **a** E and A peaks in each group. **b** Comparison of E ‘/A’ among each treatment group and the control group

BH4 and nebivolol monotherapy significantly improved SHR diastolic function: reduced LVEDP, tau, and E/E’, resulting in -dp/dtmax being more negative and increasing E’/A’ (*P* < 0.05 or *P* < 0.01, Table 3, Fig. 1).

**Table 3:** Comparison of indicators among control and experimental groups

Indexs	Control	H-BH4	L-BH4	H-N	L-N	L-N&L-BH_4_	L-N&H-BH_4_	H-N&L-BH_4_	H-N&H-BH_4_
	(*n* = 6)	(*n* = 6)	(*n* = 6)	(*n* = 6)	(*n* = 6)	(*n* = 6)	(*n* = 6)	(*n* = 6)	(*n* = 6)
Body weight g	249.8± 5.0	251.2± 5.5	248.6± 5.2	251.1± 3.5	248.6± 5.7	257.1±5.9	253.2±11.9	253.3±3.8	247.3± 2.8
Systolic BP,mm Hg	210.0±10.1	193.4±19.4	196.5±9.4	185.2±9.7^**^	190.1±23.8	185.5±16.6^*^	188.4±20.8^*^	186.±16.0^*^	185.1±11.5^*^
Heart rate, bpm	447±37	448±12^##^	436±34	347±11^*^	366±7	361±5	343±57	332±10^*#^	331±6^*##^
Diastolic measures									
E`	29.83±1.16	34.66±1.03**^##^	33.00±1.09^**##^	48.00±0.89**^##^	31.16±1.17^*##^	37.83±1.16^**##^	28.00±0.89^*##^	33.00±1.09^**##^	29.50±1.04^##^
E/E′	23.49±0.95	15.25±0.36^**^	19.65±0.63^**##^	18.63±0.45^**##^	20.45±1.40^*##^	15.43±0.75^**^	19.65±0.10^**##^	17.38±0.77^*##^	13.72±0.46^##^
E′/A′	0.77±0.03	1.19±0.03^**^	0.99±0.12^**#^	1.21±0.06^**^	1.07±0.05^**^	1.21±0.09^**^	1.03±0.07^**^	1.10±0.03^**##^	1.32±0.05^##^
Invasive measure									
Heart rate	399.1±34.2	371.3±50.2	398.4±38.6	354.0±24.1*	368.5±19.4	364.8±41.3	385.5±15.1	349.1±39.8**	353.9±33.2*
Systolic function									
Max LVSP	168.06±3.94	144.8±7.67^**^	131.81±6.09^**^	124.81±8.2^**^	139.35±4.3^**^	162.37±9.06	136.3±4.33^**^	153.59±9.1*	146.17±0.64*
+dP/dtmax, mm Hg/s	8338.20±366.75	8268.8±277.50	7257.63±73.54^##^	7724.35±107.6^##^	8279.39±246.2	8228.84±76.82	6732.±96^*##^	5212±326^**##^	4642±240**^##^
Diastolic function									
LVEDP	24.56±4.18	14.55±1.82^**^	16.2±1.21^**^	15.95±1.50^**#^	17.99±2.04^**^	14.56±1.40^**^	16.86±2.83^**^	14.89±0.52^**^	12.43±3.04^##^
-dP/dtmin,mmHg/s	-6992±123	-6780±284^##^	-6568±149^##^	-6599±287^##^	-6610±195^##^	-7753±223^*^	-6598±134^##^	-6854±224^*##^	-7787±489^*##^
-dP/dtmin/lvsp	-41.65±1.4	-46.98±4.32^*^	-49.91±2.72^**^	-53.02±4.0^**^	-47.4±0.83^*^	-47.9±3.98^*^	-48.4±2.48^**^	-40.28±3.44	-55.62±1.60
tau	38.28±2.48	25.58±0.63^**^	23.67±0.63^**^	27.84±2.05^**^	29.38±2.98^**##^	25.42±1.38^**^	22.53±2.65^**##^	25.60±0.48^**^	20.55±4.12^*##^

Notes: Values are means ± SD. WKY, Wistar-Kyoto rats; SHR, spontaneously hypertensive rat; BP, blood pressure; E = peak early diastolic LV filling velocity; A = peak late diastolic LV filling velocity; E/A = peak E and A velocity ratio; E’and A’, maximal velocity of mitral annulus during early and late diastole; LVEDd, LV end-diastolic dimension; LVSP, LV systolic pressure; tau, time constant of isovolumic LV relaxation.*represents compared with control, *P* < 0.05,**represents *P* < 0.01; # represents compared with group L-N&L-BH4, *P* < 0.05, ## represents *P* < 0.01

The following changes in SHRs diastolic function after BH4 combined with nebivolol were observed: compared with those in the placebo group, both non-invasive blood pressure and heart rate were significantly decreased in the combined treatment group (*P* < 0.05 or *P* < 0.01, Table 3). Compared with different doses of BH4 and nebivolol monotherapy group, E’/A’ in the H-N&H-BH4 combination therapy group increased significantly. In addition, LVEDP, E/E’and tau were significantly decreased in the H-N&H-BH4 combination group compared with the monotherapy group and the L-N&L-BH4 combination group, and the negative values of -dp/dtmax were more negative (*P* < 0.05 or *P* < 0.01, Table 3). The two-factor analysis of variance showed that BH4 combined with nebivolol had a significant effect on non-invasive diastolic function indicators (E/E’ and E’/A’) and invasive diastolic function indicators (tau, LVEDP, and dp/dtmin) (*P* < 0.05, Table 3). In short, BH4 combined with nebivolol had a better effect on improving diastolic dysfunction in SHRs than BH4 and nebivolol monotherapy, and the combination of high-dose BH4 and high-dose nebivolol had the most significant effect on improving diastolic dysfunction in the combined treatment groups.

### BH4 treatment significantly increased myocardial BH4 and myocardial BH4/BH2 ratio

**Fig. 2:**
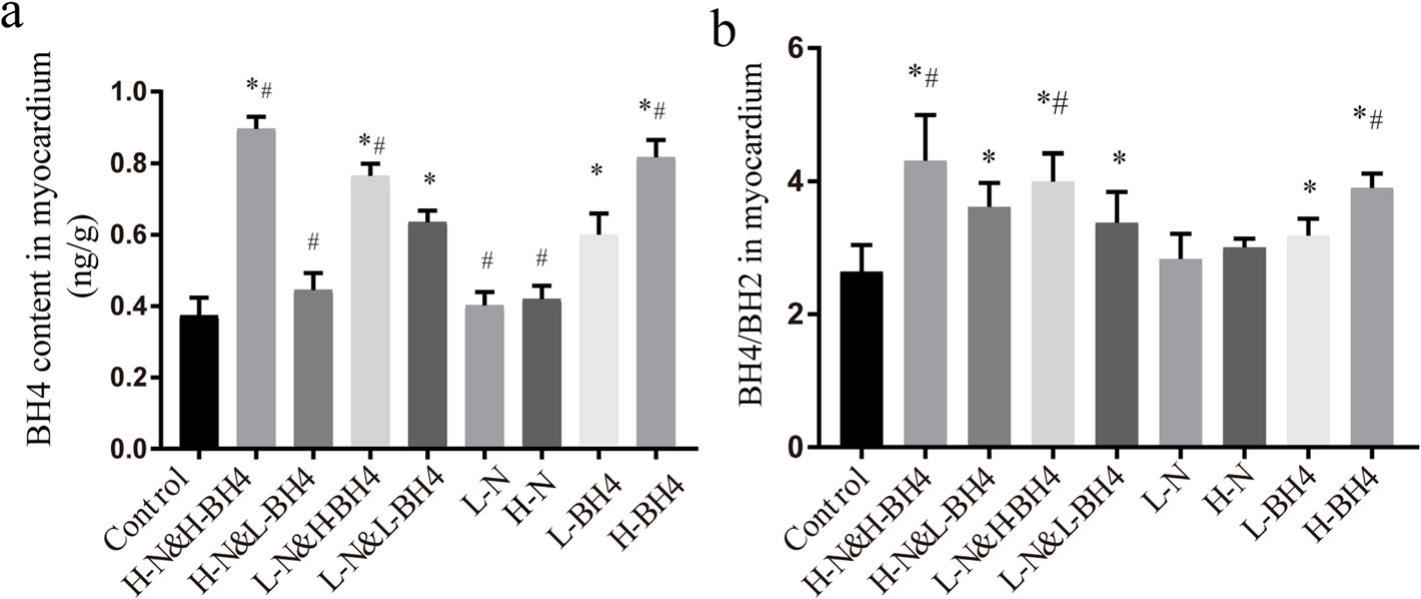
Application of HPLC to determine the content of BH4 and BH4/BH2 inmyocardium. **a** The content of BH4 in myocardium, BH4 treatment significantly increased myocardial BH4. **b** BH4/BH2 in myocardium, although nebivolol monotherapy did not improve myocardium BH4/BH2 ratio,but nebivolol combined with BH4 treatment significantly increased the BH4/BH2 ratio. *P < 0.01 versus Control, # *p* < 0.01 versus L-N&L-BH4

BH4 is an important cofactor of NOS and is necessary for NO production. Insufficient BH4 leads to the decoupling of eNOS [16], generating a large amount of reactive oxygen species, which cause oxidative stress in the corresponding organs or tissues and promote heart failure. Therefore, we used high performance liquid chromatography to detect the concentration of BH4 in each group. At the same time, since BH4 is easily oxidized to BH2 in vivo, we further detected the change of the BH4/BH2 ratio in each group. The results showed that compared with the control treatment, BH4 treatment significantly increased the level of BH4 and the ratio of BH4/BH2 in the myocardium (*P* < 0.01, Fig. 2a, b, Additional file 1). Although nebivolol monotherapy did not increase the BH4 content in the myocardium (*P* > 0.05), nebivolol combined with BH4 treatment significantly increased the BH4/BH2 ratio, especially high-dose nebivolol (5 mg/kg/d) combined with high-dose BH4 (25 mg/kg/d) group (*P* < 0.01, Fig. 2b).

### BH4 combined with nebivolol significantly inhibits oxidative stress levels

**Fig. 3:**
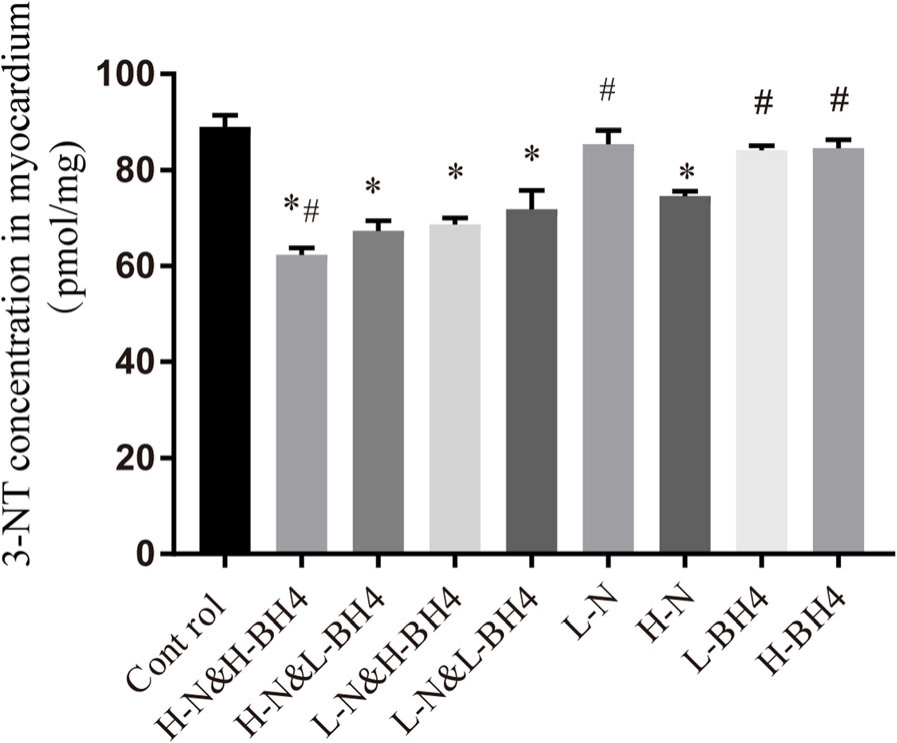
The concentration of 3-NT in myocardium. BH4 combined with nebivolol significantly inhibited oxidative stress levels. *P < 0.01 versus Control, # *p* < 0.01 versus L-N&L-BH4

3-Nitrotyrosine is a sign of oxidative stress, especially an important indicator of the reaction between NO and O_2_^−^ to form ONOO^−^. ELISA was used to detect the level of 3-nitrotyrosine in each group. The study showed a significant decrease in 3-nitrotyrosine levels after high-dose (5 mg/kg/d) nebivolol treatment alone compared to the control group (*P* < 0.01, Fig. 3), while low-dose nebivolol and different dose of BH4 alone had no significant effects (*P* > 0.05). It is worth noting that 3-nitrotyrosine levels were significantly reduced in each group after the two combined treatments, and 3-nitrotyrosine levels in the high-dose nebivolol combined with high-dose BH4 treatment groups were significantly reduced (*P* < 0.001, Fig. 3, Additional file 2), indicating BH4 and nebivolol have a synergistic effect on the downregulation of myocardial oxidative stress.

### BH4 and nebivolol can synergistically promote eNOS expression

**Fig. 4:**
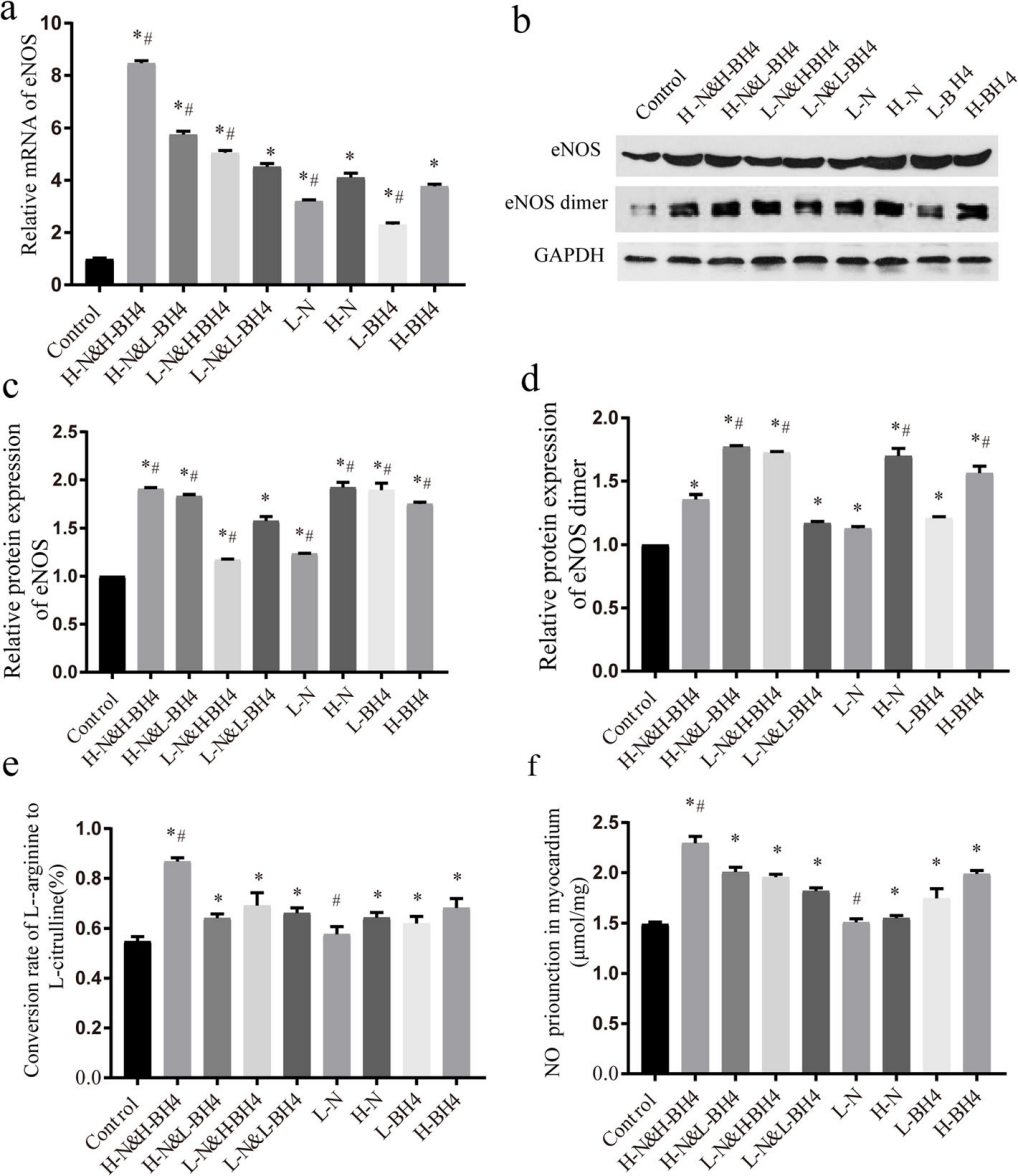
eNOS and eNOS dimer expression in myocardium. **a** The relative mRNA of eNOS in myocardium, BH4 combined with nebivolol treatment can significantly increase myocardial eNOS mRNA expression. **b** The relative protein expression of eNOS in myocardium, BH4 combined with nebivolol treatment can significantly increase the expression of eNOS protein in myocardium. **c** The relative protein expression of eNOS in myocardium. **d** The relative protein expression of eNOS dimer in myocardium. **e** eNOS activity in myocardium, high-dose BH4 combined with high-dose nebivolol can significantly increase eNOS activity. **f** The production of NO in myocardium .*P < 0.01 versus Control, # *p* < 0.01 versus L-N&L-BH4

Since the NO pathway is closely related to the level of oxidative stress in patients with diastolic heart failure, the production of NO is closely related to the activity of eNOS [17]. eNOS is essential for regulating the structure and function of blood vessel walls and is involved in the pathophysiology of cardiovascular disease. Therefore,we examined eNOS expression levels, eNOS activity, and NO production among the groups. qRT-PCR results showed that compared with the control group, BH4 or nebivolol alone could significantly increase eNOS mRNA expression, and the combination therapy was significantly more effective than the single treatment (*P* < 0.01, Fig. 4a, Additional file 3). Western blot results showed that the monotherapy group and the combination treatment group could increase eNOS total protein and eNOS dimer expression (*P* < 0.01, Fig. 4b, c, Additional file 4). Compared with the L-N & L-BH4 group, the other different dose combination treatment groups, H-BH4 and H-N group significantly enhanced eNOS dimer levels (*P* < 0.001, Fig. 4d). The isotope-labelled conversion rate of L-arginine to L-citrulline assay for myocardial eNOS activity (*P* < 0.01, Fig. 4e) and NO production test showed that the high-dose BH4 combined with high-dose nebivolol had the most significant promoting effect in each group, suggesting that the combination of nebivolol and BH4 had a significant synergistic effect on eNOS (*P* < 0.01, Fig. 4f).

### BH4 and nebivolol can synergistically promote phospholamban and SERCA2a expression

**Fig. 5:**
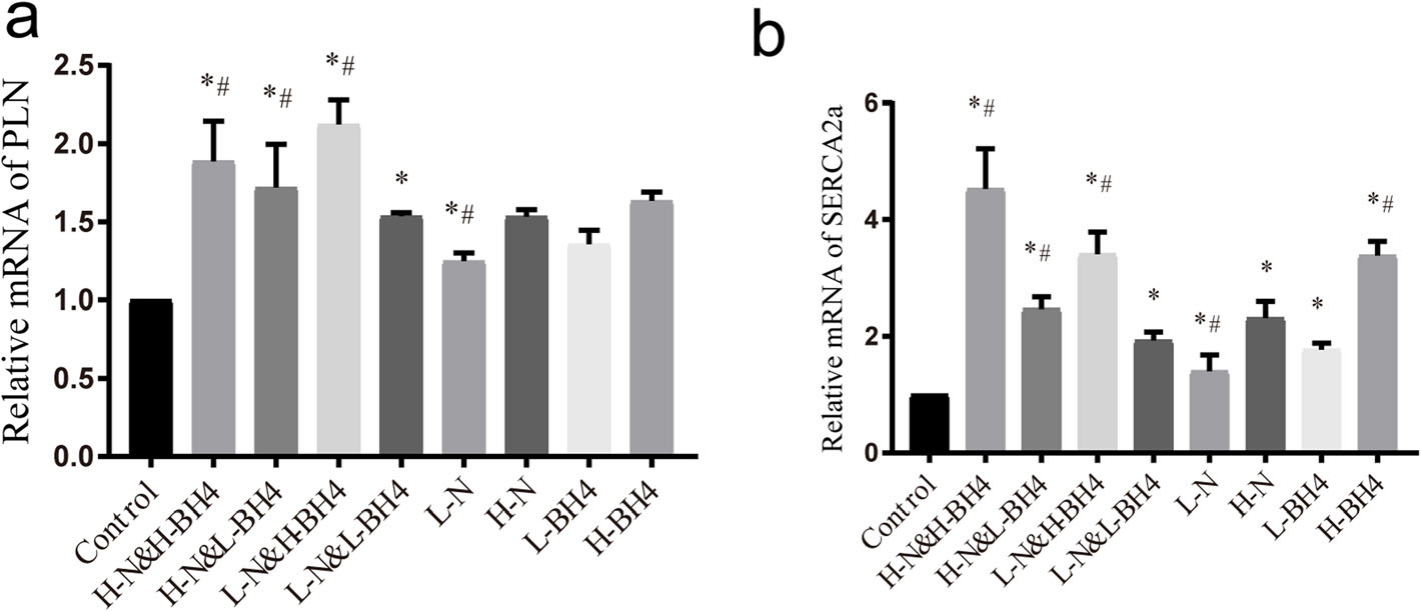
Expression of SERCA2a and PLN in each group of myocardium. **a** The relative expression of PLN mRNA in myocardium. **b** The relative expression of SERCA2a m RNA in myocardium

Left ventricular diastolic reduction is mainly related to the decreased function or reduced activity of myocardium SERCA2a, and the activity and function of SERCA2a is regulated by phosphorylation and dephosphorylation of phospholamban. Therefore, we used qRT-PCR to detect the expression levels of SERCA2a and phospholamban. The results showed that compared with the control group, BH4 and nebivolol monotherapy upregulated the expression level of phospholamban mRNA (*P* < 0.01, Fig. 5a, Additional file 5) and SERCA2a mRNA (*P* < 0.01, Fig. 5b, Additional file 6). Compared with the monotherapy group, the combined treatment group had significantly increased mRNA levels of SERCA2a and phospholamban.

### Nebivolol combined with BH4 treatment exerts protective effects on myocardium through the NO/cGMP pathway

**Fig. 6:**
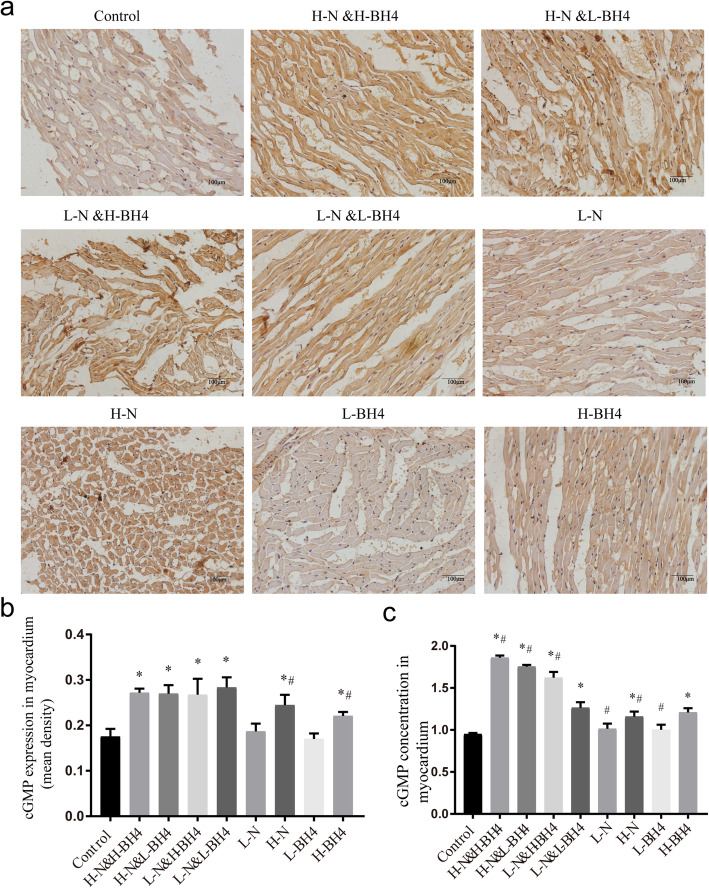
BH4 combined with Nebivolol promote the expression level of cGMP in myocardium. **a** The expression of cGMP in myocardium was detected by immunohistochemistry (*n* = 20), and the scale was 100 μm. **b** Statistics of cGMP expression in the myocardium, the data are expressed as the mean density of Image-Pro plus. **c** The expression of cGMP in myocardium was detected by ELISA.*P < 0.01 versus Control, # *p* < 0.01 versus L-N&L-BH4

NO is produced through the action of eNOS, and the production of NO activates sGC and catalyzes the formation of cGMP. Therefore, we measured the expression level of cGMP in myocardium. Immunohistochemical staining (Fig. 6a, b, Additional file 7) and ELISA (Fig. 6c) showed that compared with those in the control group, myocardial cGMP expression levels were significantly increased in all treatment groups, and cGMP expression in the high-dose nebivolol combined with high-dose BH4 treatment group was significantly higher than that in the BH4 and nebivolol monotherapy treatment group (*P* < 0.01).

## Discussion

Studies have shown that diastolic dysfunction in hypertensive rats is closely related to reduced cardiac NO production [18]. This diastolic dysfunction is reversed or improved by oral administration of BH4 [7]. The generation of NO depends on the activity of eNOS [13], and on the presence of sufficient concentration of BH4 in myocardial cells. Similarly, studies have demonstrated that nebivolol reverses the decoupling or increases the re-coupling of eNOS, increasing its activity [15]. However, it is unclear whether the combined treatment produces better protective effects.

In this study, non-invasive and invasive cardiac function tests confirmed that BH4 and nebivolol alone or in combination could reverse or prevent diastolic dysfunction. It is worth noting that the combination of nebivolol and BH4 showed a more effective protective effect. Long-term hypertension puts the heart in a state of oxidative stress, which leads to decoupling of eNOS and reduces the bioavailability of NO, eventually leading to diastolic dysfunction [15, 19]. In this study, BH4 and nebivolol combination therapy increased the level of eNOS dimer, which were active, indicating that combination therapy can increase the recoupling of eNOS and increase its activity. Furthermore, BH4 and nebivolol combination therapy significantly increased the expression of eNOS mRNA and total eNOS protein and increased eNOS activity, increasing NO production in the myocardium. It is worth mentioning that the administration of BH4 and nebivolol reduced the level of 3-NT, which is a marker of oxidative stress in the myocardium [19, 20]. This study confirms that BH4 and nebivolol have cardioprotective effects, and the mechanism is mainly related to their antioxidant capacity, increased eNOS recoupling, and NO production in the myocardium.

**Fig. 7:**
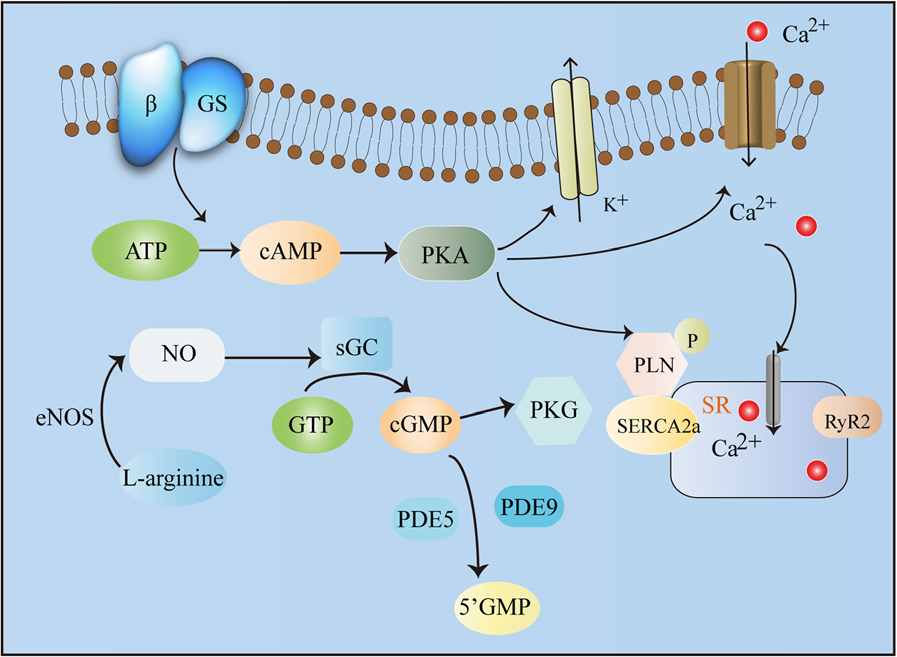
NO/cGMP pathway in cardiomyocytes. NO in the body is produced from L-arginine as a raw material, which is transported into the cell via the cell membrane arginine transporter, and is produced under the action of NOS. The production of NO then activates soluble guanylate cyclase and catalyses the formation of cGMP. The formed cGMP activates PKG. Phosphodiesterase 5 is responsible for the decomposition of cGMP produced by soluble guanylate cyclase, while Phosphodiesterase 9 is responsible for the decomposition of cGMP produced by pGC. The drug stimulates the production of a second messenger cAMP via a Gs-coupled β-adrenergic receptor. cAMP activates PKA, and PKA thenactivates K + channels also increases hyperpolarization, thus indirectly preventing extracellular Ca2 + influx and promoting vasodilation. Abbreviations: cGMP:Cyclic guanosine monophosphate; eNOS: Endothelial nitric oxide synthase; NOS: Nitric oxide synthase; PKA: cAMP-dependent protein kinase; PKG: cGMP-dependent protein kinase; 5’GMP:Guanosine 5′-monophosphate; SERCA2a: Sarco/endoplasmic reticulum Ca2 + −ATPase, SR: Sarco/endoplasmicreticulum, RyR2: Ryanodine receptor 2

NO in the body is produced from L-arginine as a raw material, which is transported into the cell via the cell membrane arginine transporter, and is produced under the action of NOS. Subsequently, the production of NO activates sGC and catalyses the formation of cGMP [21]. Abdallah et al. showed that cGMP increased the reuptake and storage of Ca2+ in the endoplasmic reticulum because of increased SERCA2a activity [22]. However, SERCA2a activity is closely related to phospholamban phosphorylation (Fig. 7) [23]. Li et al. found that compared with those in the WKY rats group with normal blood pressure, the Na + −Ca2+ exchanger and phospholamban proteins in SHRs myocardial cells increased significantly, while SERCA2a decreased significantly, which confirmed that due to abnormal SR and cell membrane processing of Ca2+ in hypertension, the systolic function was changed [24]. This study showed that BH4 and/or nebivolol increased the expression of phospholamban and SERCA2α in the myocardium. In particular, the high-dose nebivolol combined with high-dose BH4 treatment group had a more significant effect than the monotherapy group.

Fang et al. confirmed that nebivolol treatment can improve left ventricular diastolic function by increasing the bioavailability of NO, and L-NNA, an inhibitor of NOS, antagonizes this protective effect [25]. This study showed that nebivolol can reduce oxidative stress and re-couple eNOS to increase NO production. The increase in heart rate is an important determinant of myocardial oxygen consumption. Studies of the effect of nebivolol and other drugs on myocardial oxygen consumption in patients with hypertension found that the use of nebivolol alone can decrease the heart rate and reduce myocardial oxygen demand, while also increasing the myocardial oxygen supply [26, 27]. However, there was no significant difference in systolic blood pressure or heart rate between the combination therapy group and the nebivolol monotherapy group. This indirectly indicates that the synergistic effect of the combined treatment of BH4 and nebivolol is independent of the effect of nebivolol on blood pressure and heart rate reduction, which is related to the NO pathway.

This study confirmed that BH4 and nebivolol had a synergistic protective effect on heart failure. In this study, invasive and non-invasive cardiac function indicators showed that compared with nebivolol and BH4 monotherapy, the combined application of nebivolol and BH4 can significantly improve the diastolic function of the heart. The combination therapy had a stronger effect on increasing BH4/BH2, as well as increasing the production of eNOS-derived NO. At the same time, the combined treatment group more significantly reduced 3-NT levels, and the high-dose nebivolol combined with the high-dose BH4 treatment group had a more significant effect. Moreover, the expression of cGMP, which is an effector of NO, was significantly increased in the combination treatment group compared with the control group and the monotherapy group.

This study provided a basis for the combined application of nebivolol and BH4 in the treatment of diastolic dysfunction in hypertension. In future investigations, the mechanisms need to be further clarified, and the effects of combined treatment on the expression of other subtypes of NOS can be detected. Immunohistochemical staining and western blot can be used to detect the expression of β1-AR, β2-AR, and β3-AR in myocardial tissue.

Study found that the possible mechanisms by which BH4 and nebivolol have a protective effect on diastolic function are as follows: 1. BH4 and/or nebivolol can maintain a relatively high BH4/BH2 ratio. 2. Nebivolol reduces oxidative stress in hypertensive myocardium through anti-oxidation, while BH4 has a weak effect in this respect. 3. Nebivolol cooperates with BH4 to increase eNOS recoupling to exert protective effects. 4. Activation of eNOS increases cGMP expression and activates SERCA2a by increasing PLN phosphorylation. 5. Activation of SERCA2a increases SR reuptake of Ca2+, thereby improving diastolic capacity. There are certain limitations in this study. Nebivolol is a β-receptor blocker. In this study, NOS inhibitors, β3-AR inhibitors and agonists were not used in a controlled study to further confirm its mechanism. There are some limitations in this study. Nebivolol is a β-receptor blocker. NOS inhibitors, β3-AR inhibitors or agonists were not used in this study to further confirm its mechanism.

The results of this study indicate that the combination of nebivolol and BH4 is more conducive to improving diastolic dysfunction and can be used as a new treatment strategy.

## Conclusions

In summary, we confirmed that BH4 and nebivolol have their own advantages and disadvantages in the treatment of diastolic dysfunction caused by hypertension, and combined treatments can complement each other and play a synergistic protective role through the NO/cGMP signalling pathway.

## Supplementary Information

**Additional file 1.**

**Additional file 2.**

**Additional file 3.**

**Additional file 4: Figure 1.** Western blot was used to detect the protein expression of eNOS in myocardium (Fig. 4b in the manuscript). **Figure 2.** Western blot was used to detect the protein expression of eNOS dimer in myocardium (Fig. 4b in the manuscript). **Figure 3.** Western blot was used to detect the protein expression of GAPDH in myocardium (Fig. 4b in the manuscript).

**Additional file 5.**

**Additional file 6.**

**Additional file 7.**

## Data Availability

The datasets used and/or analyzed during the current study are available from the corresponding author on reasonable request.

## Abbreviations

ANOVA: One-way analysis of variance
A: Peak late diastolic LV filling velocity
β-AR: β-adrenergic receptor
cGMP: Cyclic guanosine monophosphate
eNOS: Endothelial nitric oxide synthase
BH4: Tetrahydrobiopterin
E’and A’: Maximal velocity of mitral annulus during early and late diastole
E/A: Peak E and A velocity ratio
E: Peak early diastolic LV filling velocity
LVEDP: LV end-diastolic pressure
tau: Time constant of isovolumic LV relaxation
NO: Nitric oxide
NOS: Nitric oxide synthase
qRT-PCR: Quantitative real-time PCR
SERCA2a: Sarcoplasmic reticulum Ca^2+^-ATPase 2a
SHRs: Spontaneously hypertensive rats
WKY: Wistar-Kyoto
