# Supplementary material for: Nebivolol combined with tetrahydrobiopterin affects diastolic function in spontaneously hypertensive rats via the nitric oxide/cyclic guanosine monophosphate signalling pathway
Source: BMC Pharmacol Toxicol. 2020 Dec 2;21:84. doi: 10.1186/s40360-020-00460-z (PMC7709331; doi:10.1186/s40360-020-00460-z)
Supplement: Supplementary file 4 — Additional file 4: Figure 1. Western blot was used to detect the protein expression of eNOS in myocardium (Fig. 4b in the manuscript). Figure 2. Western blot was used to detect the protein expression of eNOS dimer in myocardium (Fig. 4b in the manuscript). Figure 3. Western blot was used to detect the protein expression of GAPDH in myocardium (Fig. 4b in the manuscript). [file 40360_2020_460_MOESM4_ESM.doc]

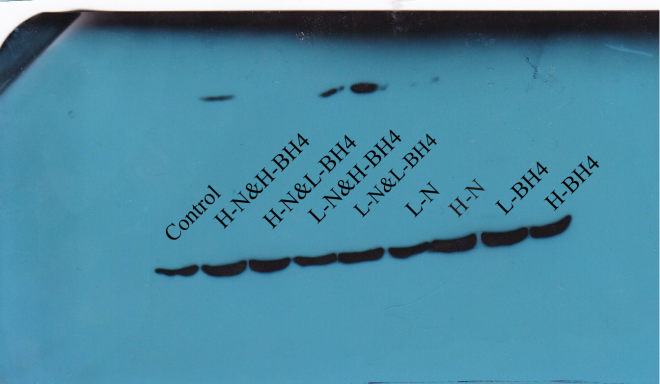


**Figure 1** Western blot was used to detect the protein expression of eNOS in myocardium (Figure 4b in the manuscript).


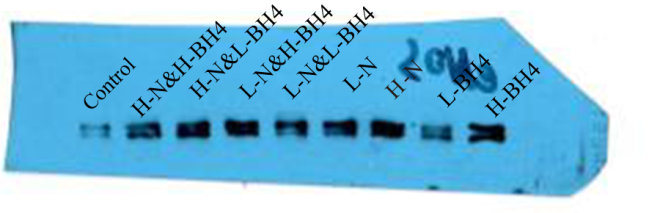


**Figure 2**  Western blot was used to detect the protein expression of eNOS dimer in myocardium (Figure 4b in the manuscript).


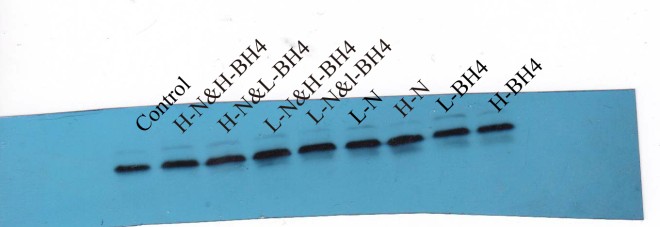


**Figure 3**  Western blot was used to detect the protein expression of GAPDH in myocardium (Figure 4b in the manuscript).
